# Supplementary material for: Low-Dose Oral Iron Replacement Therapy Is Effective for Many Japanese Hemodialysis Patients: A Retrospective Observational Study
Source: Nutrients. 2022 Dec 27;15(1):125. doi: 10.3390/nu15010125 (PMC9824721; doi:10.3390/nu15010125)
Supplement: Supplementary file 1 [file nutrients-15-00125-s001.zip › nutrients-2099520-supplementary.pdf]

nutrients

MDPI

Supplementary Materials

Low-Dose Oral Iron Replacement Therapy Is Effective for Many Japanese Hemodialysis Patients: A Retrospective Observational Study

Chie Ogawa [1,2,*], Ken Tsuchiya [2,3], Mineko Kanemitsu [1] and Kunimi Maeda [1,2]

Table S1. One-way repeated measures analysis of variance was performed for changes over time.

		3–4 week	7–8 week	11–12 week	15–16 week	19–20 week	21–24 week	27–28 week
Hb	①	*	*	*	*	*	*	*
	②			**	*	*	*	*
	③	**	*	*				
RBC	①	*	*	*	*	**		
	②				*			
	③		*	**				**
MCH	①			**	*	*	*	*
	②			**	*	*	*	*
	③		**	**	*	*	*	*
s-ft	①	*	*	*	*	*	*	*
	②				**	*		**
	③	*	*	*	*	*	*	*
TSAT	①	*	*	*	*	*	*	*
	②				**	**		
	③				*	**	*	**

①: group1, ②: group2, ③: group3.
*; $p < 0.01$, **; $p < 0.05$ (vs. 0 wk).
Hb; Hemoglobin, RBC; Red blood cells, MCH; mean corpuscular hemoglobin, s-ft; serum ferritin, TSAT; Transferrin saturation.
